# Machine learning-driven SLC prognostic signature for glioma: predicting survival and immunotherapy response

**DOI:** 10.3389/fphar.2025.1585639

**Published:** 2025-06-06

**Authors:** Jianghua Lin, Xiao Yang, Kaijun Zhao, Yu’e Liu

**Affiliations:** ^1^ Department of Neurosurgery, Shanghai East Hospital, School of Medicine, Tongji University, Shanghai, China; ^2^ Department of Hematology/Oncology, Boston Children’s Hospital, Harvard Medical School, Boston, MA, United States

**Keywords:** glioma, SLC family, machine learning, immunotherapy, prognostic biomarker

## Abstract

**Introduction:**

Gliomas are the most common and aggressive primary brain tumors, characterized by significant heterogeneity and poor prognosis. Despite advancements in treatment, therapeutic resistance and tumor recurrence remain major challenges. Identifying novel molecular biomarkers is essential for improving prognosis and developing more effective therapies.

**Methods:**

In this study, we developed a solute carrier family prognostic signature (SLCFPS) for gliomas using univariate Cox regression and machine learning algorithms across five independent glioma cohorts. Prognostic performance was evaluated through Kaplan-Meier survival analysis, concordance index (C-index), and receiver operating characteristic (ROC) curve analysis. The immune landscape, immunotherapy response, and drug sensitivity were further analyzed using bioinformatics tools such as ESTIMATE, xCell, TIDE, and drug response correlation analysis.

**Results:**

SLCFPS effectively stratified glioma patients into high- and low-risk groups, with higher scores associated with poorer survival outcomes. The model demonstrated superior predictive performance compared to existing glioma prognostic models. Additionally, SLCFPS was linked to an immunosuppressive tumor microenvironment and upregulated immune checkpoints, indicating potential implications for immunotherapy response. Furthermore, SLCFPS correlated with drug sensitivity, suggesting potential therapeutic options for glioma treatment.

**Discussion:**

Our findings highlight SLCFPS as a robust biomarker for glioma prognosis and treatment response. By providing insights into tumor immunity, this model may aid in the development of personalized therapeutic strategies. Further validation in clinical settings is necessary to explore its full potential in guiding glioma management.

## 1 Introduction

Gliomas are the most common and aggressive primary tumors of the central nervous system, exhibiting substantial heterogeneity in their molecular characteristics and clinical outcomes (Schaff and Mellinghoff, 2023). Despite advances in surgical resection, radiotherapy, and chemotherapy, the prognosis for glioblastoma (GBM), the most malignant subtype, remains poor, with a median overall survival of approximately 15 months. The identification of reliable prognostic biomarkers is crucial for improving risk stratification, guiding therapeutic decisions, and ultimately enhancing patient outcomes. In recent years, molecular classification has significantly contributed to our understanding of glioma biology (Verdugo et al., 2022). Key genetic alterations, including mutations in isocitrate dehydrogenase (IDH1/2), 1p/19q co-deletion, and O6-methylguanine-DNA methyltransferase (MGMT) promoter methylation, have been incorporated into clinical practice to refine prognostic predictions (Lan et al., 2024). Other biomarkers related to immunotherapy or cancer metabolism may indicate treatment efficacy and hold potential utility in certain gliomas (Liu et al., 2023a; Liu et al., 2023b; Liu et al., 2023c). However, these markers alone fail to fully capture the complexity of glioma progression, highlighting the need for novel prognostic models that integrate multiple molecular and clinical parameters.

The solute carrier (SLC) family constitutes one of the largest groups of transmembrane transporters, encompassing over 400 members that facilitate the transport of nutrients, ions, and metabolites across cellular membranes (Sun et al., 2024; Hu et al., 2020). Increasing evidence has highlighted the involvement of SLC proteins in cancer biology, where they contribute to tumor progression, metabolic reprogramming, immune evasion, and therapeutic resistance (Wu et al., 2021). For example, the glutamine transporter SLC1A5 and SLC7A11 regulate glutamine metabolism and redox balance, respectively, thereby supporting tumor cell survival and resistance to oxidative stress (Yoo et al., 2020; Wang et al., 2021). Additionally, SLC transporters have been linked to immune regulation, affecting T cell function and tumor-associated macrophage polarization. The itaconate transporter SLC13A3 impairs tumor immunity by conferring ferroptosis resistance (Lin et al., 2024). The creatine transporter SLC6A8 promotes macrophage polarization from the M1 to M2 phenotype by differentially modulating cytokine-driven signaling pathways (Ji et al., 2019). In glioma, sodium/hydrogen exchanger 1 (NHE1), encoded by the SLC9A1 gene has been studied as a marker tumorigenesis and prognosis (Guan et al., 2018). The NHE1 inhibitor HOE642 reduced glioma growth and invasion. This effect was associated with an enhanced immunogenic tumor microenvironment, characterized by increased CD8^+^ T-cell accumulation, upregulated interferon-gamma expression, and improved response to anti-PD-1 therapy (Guan et al., 2018). Despite these findings, a comprehensive prognostic model based on SLC family genes has yet to be established for glioma. Understanding the prognostic significance of SLC transporters could provide valuable insights into glioma biology and offer novel therapeutic strategies for improving patient outcomes.

In this study, we developed a robust SLC family prognostic signature (SLCFPS) for glioma using multi-cohort survival analysis and machine learning-based modeling. We identified prognostic SLC genes across five independent cohorts and constructed an optimized predictive model, which was validated in multiple datasets. Beyond prognosis, we explored the biological and clinical relevance of SLCFPS, including its association with the tumor immune microenvironment, immune checkpoint expression, and tumor purity. Furthermore, we assessed its potential as a biomarker for immunotherapy response and drug sensitivity. Our findings highlight SLCFPS as a powerful prognostic tool, offering insights into glioma immunity and treatment response, with implications for personalized therapeutic strategies.

## 2 Materials and methods

### 2.1 Identification of prognostic SLC genes

Univariate Cox regression analysis was performed using the “survival” package in R on five glioma cohorts to assess the prognostic significance of solute carrier (SLC) family genes. Genes with a p-value < 0.05 were considered statistically significant. To identify common prognostic SLC genes across cohorts, a Venn diagram intersection analysis was conducted using the “VennDiagram” package, ultimately selecting 64 candidate SLC genes.

### 2.2 Machine learning-based model construction

To develop an optimal prognostic model, a total of 10 different machine learning algorithmic combinations were applied to the 64 selected genes, including CoxBoost, elastic net (Enet), generalized boosted regression modeling (GBM), least absolute shrinkage and selection operator (Lasso), partial least squares regression for Cox (plsRCox), random survival forest (RSF), Ridge regression, supervised principal components (SuperPC), stepwise Cox, and survival support vector machine (survival-SVM) (Xiao et al., 2024). Each model’s predictive performance was assessed using the concordance index (C-index) to determine accuracy and robustness. All of the above methods were implemented using the “Mime1” package (Liu et al., 2024).

### 2.3 Selection of the optimal predictive model

A comprehensive evaluation of 101 different machine learning algorithm combinations was conducted across the five glioma cohorts. The model achieving the highest average C-index was selected as the final prognostic framework.

### 2.4 Performance validation of the predictive model

The predictive accuracy of the final model—Stepwise Cox regression (StepCox [forward]) combined with Elastic Net regularization [Enet (α = 0.1)]—was assessed at 1-year, 3-year, and 5-year survival time points. Stepwise Cox regression was implemented using the “stepAIC” function from the “MASS” package, while Elastic Net regularization was performed using the “glmnet” package (with α = 0.1). Time-dependent receiver operating characteristic (ROC) curve analysis was performed across all five cohorts using the “timeROC” package, and area under the curve (AUC) values were calculated to validate its prognostic utility.

### 2.5 Survival analysis of SLCFPS

To determine the prognostic significance of the SLC family prognostic signature (SLCFPS), patients from five independent glioma cohorts (TCGA_GBMLGG, CGGA_325, CGGA_693, Rembrandt, and Gravendeel) were stratified into high- and low-risk groups based on their SLCFPS scores. The SLCFPS score is calculated based on the expression levels of selected genes, with weights assigned according to the coefficients derived from our prognostic model. For risk stratification, the median value of the SLCFPS scores was used as the cutoff point to divide patients into high- and low-risk groups. Kaplan-Meier survival analysis was performed using the “survival” package to compare overall survival (OS) between the two groups, and statistical significance was determined using the “survminer” package for the log-rank test.

### 2.6 Comparative analysis of predictive performance

To compare the prognostic performance of SLCFPS against conventional clinical and pathological features, the “survival” package was used to calculate the concordance index (C-index) for each prognostic factor across the five cohorts. The predictive accuracy of SLCFPS was then compared to standard clinical parameters, including age, tumor grade, and molecular markers, using the “pec” package to assess its relative prognostic value.

### 2.7 Univariate Cox regression analysis

To evaluate whether SLCFPS serves as an independent prognostic factor, univariate Cox proportional hazards regression analysis was conducted in each cohort using the “survival” package. Hazard ratios (HRs) with corresponding 95% confidence intervals (CIs) and p-values were calculated using the coxph function in the “survival” package to determine the statistical significance of SLCFPS in predicting patient survival outcomes.

### 2.8 Validation of SLCFPS in other cancer types

To investigate the generalizability of SLCFPS beyond glioma, its prognostic value was assessed in six additional cancer types from The Cancer Genome Atlas (TCGA), including cervical squamous cell carcinoma and endocervical adenocarcinoma (CESC), kidney renal clear cell carcinoma (KIRC), kidney renal papillary cell carcinoma (KIRP), acute myeloid leukemia (LAML), pancreatic adenocarcinoma (PAAD), and sarcoma (SARC). Patients were stratified into high- and low-risk groups based on their SLCFPS scores, and Kaplan-Meier survival analysis was performed using the “survival” package to compare OS between the two groups, with statistical significance determined by the “survminer” package via the log-rank test.

To assess the predictive performance of SLCFPS in these cancer types, time-dependent ROC curve analyses were conducted using the “timeROC” package for each cohort. The prognostic accuracy of the Stepwise Cox regression + Elastic Net [Enet (α = 0.1)] model was evaluated using AUC values at different time points.

### 2.9 Comparison with other glioma prognostic signatures

To evaluate the prognostic robustness of SLCFPS in comparison with previously published glioma signatures, we retrieved multiple glioma prognostic models from the literature. The hazard ratio (HR) of SLCFPS was compared with these signatures across independent glioma cohorts using the “cal_RS_pre.prog.sig” function from the “Mime1” package, while the concordance index (C-index) was compared using the “cal_cindex_pre.prog.sig” function in the same package. Statistical significance was assessed using the wilcox.test function in the “stats” package.

### 2.10 Enrichment pathway analysis

To investigate the biological pathways associated with SLCFPS, Gene Set Variation Analysis (GSVA) was conducted using the Hallmark gene sets. Samples were stratified into high and low SLCFPS expression groups, and pathway enrichment scores were compared between these groups. Additionally, Gene Ontology (GO) enrichment analysis was performed to identify the top five significantly enriched pathways in the high- and low-SLCFPS groups, respectively. The enrichment analysis was conducted using the “clusterProfiler” R package, and pathways with adjusted *p*-values < 0.05 were considered statistically significant.

### 2.11 Immune landscape analysis

To explore the immune microenvironment characteristics of high- and low-SLCFPS groups, immune scores were computed using the “xCell” and “ESTIMATE” algorithms across five glioma cohorts, with the “IOBR” package used to perform both the “xCell” and “ESTIMATE” analyses (Zeng et al., 2021). Differences in immune scores between high- and low-SLCFPS groups were assessed using the wilcox.test function in the “stats” package. Additionally, tumor purity was estimated using the “ESTIMATE” method, and its correlation with SLCFPS scores was calculated using the cor function in the “stats” package.

To further analyze the immune checkpoint landscape, expression levels of key immune checkpoint molecules were compared between high- and low-SLCFPS groups using the wilcox.test function in the “stats” package. Statistical significance was denoted as *p* < 0.05 (*), *p* < 0.01 (**), *p* < 0.001 (***).

### 2.12 Prediction of immunotherapy response

To assess the predictive value of SLCFPS for immunotherapy response, the TIDE algorithm was applied to estimate the likelihood of immune therapy response in high- and low-SLCFPS groups (Fu et al., 2020). The percentage of predicted responders and non-responders was compared across multiple glioma cohorts. Additionally, Kaplan-Meier survival analysis was conducted in the PRJNA482620 immunotherapy cohort using the “survival” package to evaluate whether SLCFPS expression was associated with patient survival following immune checkpoint blockade therapy. Statistical significance was assessed using the “survminer” package via the log-rank test, and differences in TIDE scores were analyzed using the wilcox.test function in the “stats” package.

### 2.13 Drug sensitivity analysis

To explore the relationship between SLCFPS and drug sensitivity, drug response scores for multiple small-molecule inhibitors and the corresponding expression data of cell lines were obtained from the Genomics of Drug Sensitivity in Cancer (GDSC) database using the “drugSensitivity” package (Yang et al., 2013). These data were used as a training set, and the “oncoPredict” R package was utilized to predict drug response scores for five glioma cohorts (Maeser et al., 2021).The correlation between SLCFPS scores and drug response scores was calculated using Spearman’s correlation analysis, with the cor function from the “stats” package, and a correlation coefficient threshold of < −0.5 indicating a potential association with drug sensitivity. Differences in drug response scores between high- and low-SLCFPS groups were analyzed using the wilcox.test function from the “stats” package.

## 3 Results

### 3.1 Development and validation of the SLC family prognostic signature (SLCFPS)

**FIGURE 1:**
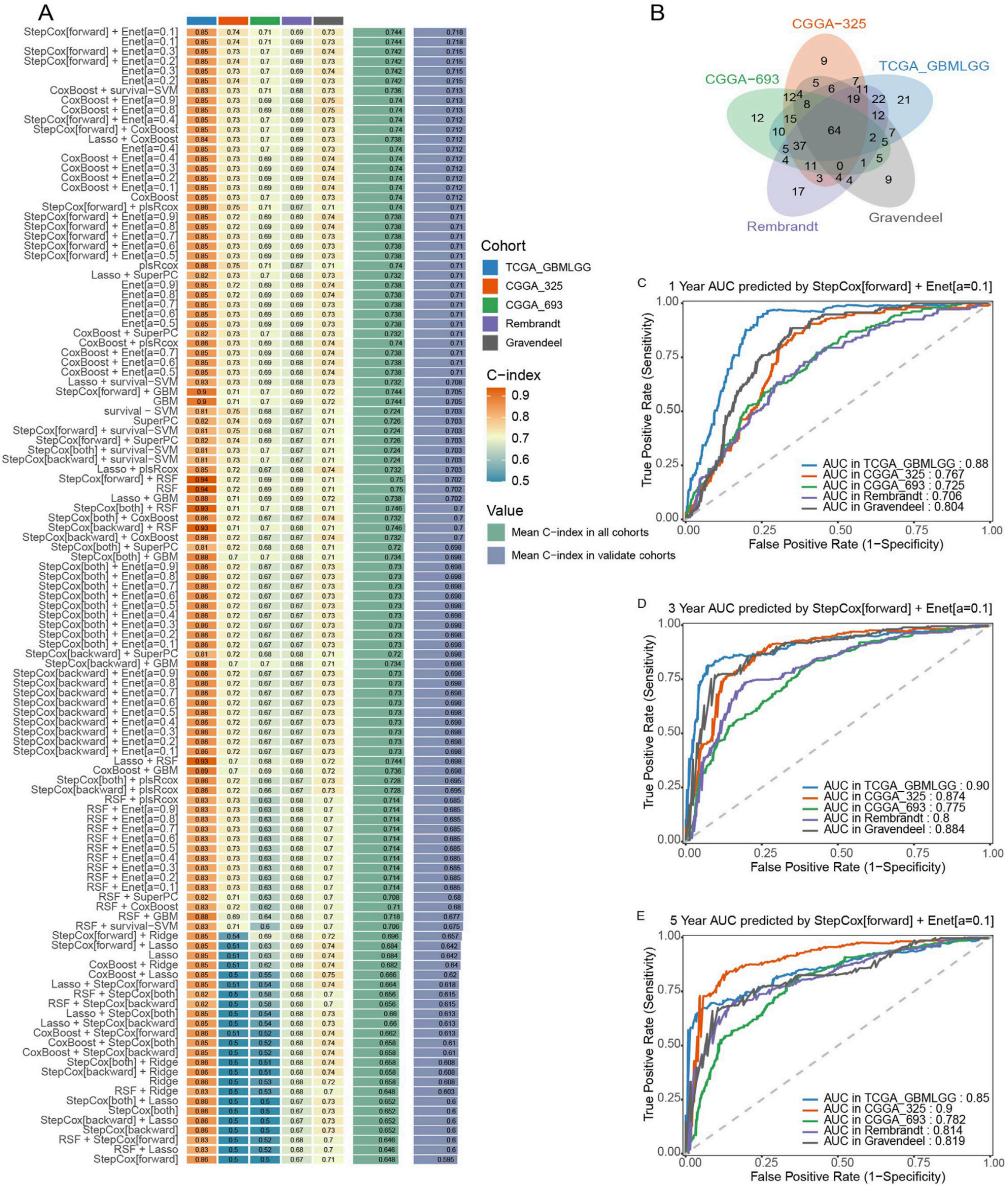
Construction and testing of the SLC Family prognostic signature (SLCFPS) **(A)** C-index of 101 machine learning algorithm combinations across five glioma cohorts. **(B)** Univariate Cox regression analysis was used on five glioma cohorts to identify SLC family genes, followed by Venn diagram intersection analysis. **(C)** One-year survival AUC curves of the StepCox [forward] + Enet [alpha = 0.1] model across five glioma cohorts. **(D)** Three-year survival AUC curves of the StepCox [forward] + Enet [alpha = 0.1] model across five glioma cohorts. **(E)** Five-year survival AUC curves of the StepCox [forward] + Enet [alpha = 0.1] model across five glioma cohorts.

To construct a robust prognostic model based on the SLC gene family, we performed a multi-step computational analysis integrating survival statistics and machine learning-based predictive modeling across five independent glioma cohorts. First, univariate Cox regression analysis was conducted on each cohort to identify SLC genes significantly associated with patient prognosis (*p* < 0.05). A total of 64 prognostic SLC genes were identified through intersection analysis using a Venn diagram (Figure 1B). Next, we employed a machine learning-based approach to develop the optimal prognostic model. A total of 10 algorithms were tested in different combinations to construct the predictive framework. To ensure robustness, we further evaluated the concordance index (C-index) across 101 machine learning algorithm combinations in all five glioma cohorts (Figure 1A). The model achieving the highest average C-index was selected as the final predictive model. To assess the prognostic performance of the selected model, we applied time-dependent receiver operating characteristic (ROC) curve analysis at 1-year, 3-year, and 5-year survival time points across all five glioma cohorts. The Stepwise Cox regression (StepCox[forward]) combined with Elastic Net regularization [Enet (α = 0.1)] demonstrated stable and high predictive accuracy, as shown by the AUC values at each time point (Figures 1C–E). Together, these findings indicate that the SLCFPS model effectively stratifies glioma patients based on prognosis, providing a potential tool for risk assessment and personalized treatment strategies.

### 3.2 Survival analysis and predictive performance assessment of SLCFPS

**FIGURE 2:**
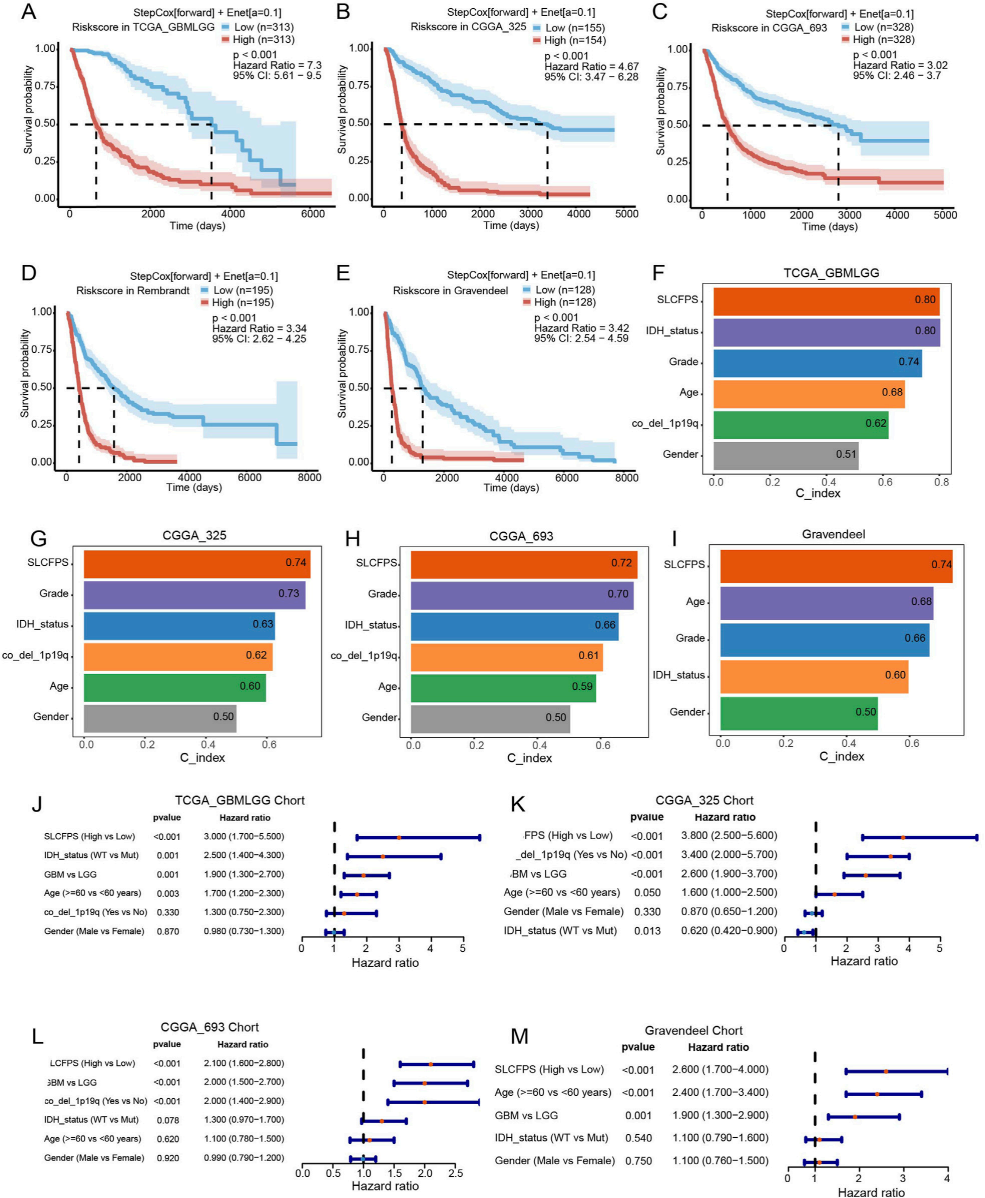
Survival analysis and predictive performance assessment of SLCFPS. **(A)** Overall survival (OS) of high and low SLCFPS groups in the TCGA_GBMLGG cohort. **(B)** OS of high and low SLCFPS groups in the CGGA_325 cohort. **(C)** OS of high and low SLCFPS groups in the CGGA_693 cohort. **(D)** OS of high and low SLCFPS groups in the Rembrandt cohort. **(E)** OS of high and low SLCFPS groups in the Gravendeel cohort. **(F)** Comparison of the C-index between clinical pathological features and SLCFPS in the TCGA_GBMLGG cohort. **(G)** Comparison of the C-index between clinical pathological features and SLCFPS in the CGGA_325 cohort. **(H)** Comparison of the C-index between clinical pathological features and SLCFPS in the CGGA_693 cohort. **(I)** Comparison of the C-index between clinical pathological features and SLCFPS in the Gravendeel cohort. **(J)** Univariate analysis of clinical pathological features and SLCFPS in the TCGA_GBMLGG cohort. **(K)** Univariate analysis of clinical pathological features and SLCFPS in the CGGA_325 cohort. **(L)** Univariate analysis of clinical pathological features and SLCFPS in the CGGA_693 cohort. **(M)** Univariate analysis of clinical pathological features and SLCFPS in the Gravendeel cohort.

To evaluate the prognostic significance of the SLCFPS, we conducted survival analysis across five independent glioma cohorts. Patients were stratified into high- and low-risk groups based on SLCFPS scores, and Kaplan-Meier survival curves were generated to compare overall survival (OS) among these groups. The results demonstrated that in all five cohorts (TCGA_GBMLGG, CGGA_325, CGGA_693, Rembrandt, and Gravendeel) patients in the high-SLCFPS group exhibited significantly worse OS compared to those in the low-SLCFPS group (Figures 2A–E), confirming the robust prognostic value of the SLCFPS model. Next, to assess the predictive power of SLCFPS in comparison to traditional clinical and pathological features, we computed and compared the concordance index (C-index) across the five cohorts. In the TCGA_GBMLGG (Figure 2F), CGGA_325 (Figure 2G), CGGA_693 (Figure 2H), and Gravendeel (Figure 2I) cohorts, SLCFPS outperformed or was comparable to standard clinical parameters, highlighting its superior prognostic capability. Furthermore, univariate Cox regression analysis was performed to determine the independent prognostic relevance of SLCFPS compared to other clinical variables. Across all datasets, SLCFPS remained a significant prognostic factor, with hazard ratios indicating a strong correlation between higher SLCFPS scores and poorer survival outcomes (Figures 2J–M). These findings underscore the clinical relevance of SLCFPS as an independent and robust prognostic biomarker for glioma patients.

### 3.3 Prognostic value of SLCFPS in other cancer types

**FIGURE 3:**
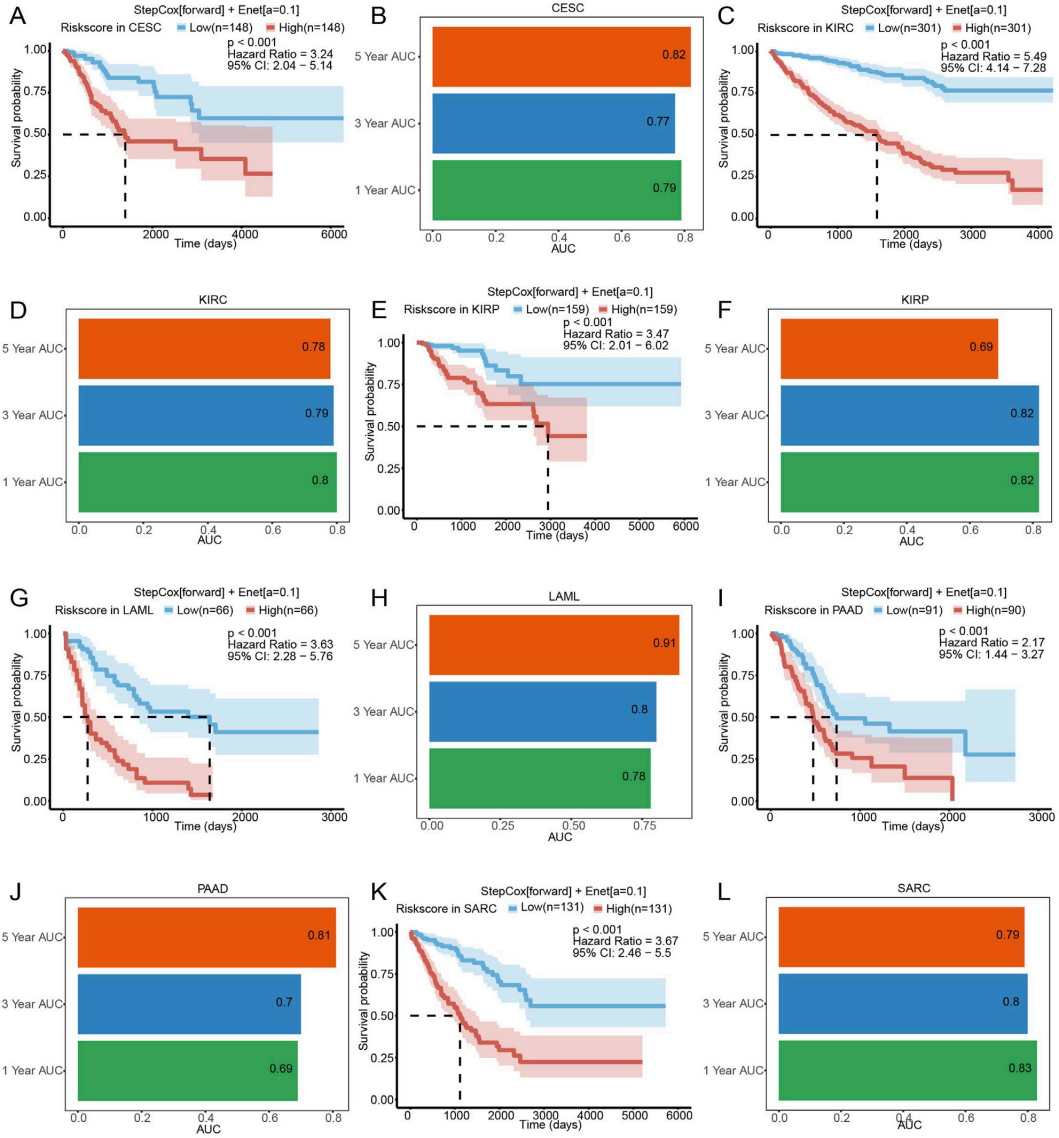
The predictive characteristics of SLCFPS in cohorts of other cancers. **(A)** OS of high and low SLCFPS groups in the TCGA_CESC cohort. **(B)** Time-dependent ROC curves of the StepCox [forward] + Enet [alpha = 0.1] model in the TCGA_CESC cohort. **(C)** OS of high and low SLCFPS groups in the TCGA_KIRC cohort. **(D)** Time-dependent ROC curves of the StepCox [forward] + Enet [alpha = 0.1] model in the TCGA_KIRC cohort. **(E)** OS of high and low SLCFPS groups in the TCGA_KIRP cohort. **(F)** Time-dependent ROC curves of the StepCox [forward] + Enet [alpha = 0.1] model in the TCGA_KIRP cohort. **(G)** OS of high and low SLCFPS groups in the TCGA_LAML cohort. **(H)** Time-dependent ROC curves of the StepCox [forward] + Enet [alpha = 0.1] model in the TCGA_LAML cohort. **(I)** OS of high and low SLCFPS groups in the TCGA_PAAD cohort. **(J)** Time-dependent ROC curves of the StepCox [forward] + Enet [alpha = 0.1] model in the TCGA_PAAD cohort. **(K)** OS of high and low SLCFPS groups in the TCGA_SARC cohort. **(L)** Time-dependent ROC curves of the StepCox [forward] + Enet [alpha = 0.1] model in the TCGA_SARC cohort.

To assess the broader applicability of SLCFPS modal beyond glioma, we evaluated its prognostic significance across six additional cancer types using The Cancer Genome Atlas (TCGA) cohorts. Patients in each cohort were stratified into high- and low-risk groups based on their SLCFPS scores, and Kaplan-Meier survival analysis was performed to compare overall survival (OS) between the two groups. The results demonstrated that a higher SLCFPS score was significantly associated with poorer OS in multiple cancer types, including cervical squamous cell carcinoma and endocervical adenocarcinoma (CESC) (Figure 3A), kidney renal clear cell carcinoma (KIRC) (Figure 3C), kidney renal papillary cell carcinoma (KIRP) (Figure 3E), acute myeloid leukemia (LAML) (Figure 3G), pancreatic adenocarcinoma (PAAD) (Figure 3I), and sarcoma (SARC) (Figure 3K). In each cohort, the log-rank test confirmed a statistically significant survival difference between the high- and low-risk groups (p < 0.05). To further evaluate the predictive performance of SLCFPS in these cancers, we conducted time-dependent receiver operating characteristic (ROC) analyses. The Stepwise Cox regression (StepCox[forward]) + Elastic Net (Enet[α = 0.1]) model exhibited robust predictive power in each cohort, as indicated by the area under the curve (AUC) values across different time points (Figures 3B, D, F, H, J, L). These findings suggest that SLCFPS has generalizable prognostic utility across multiple malignancies, reinforcing its potential as a valuable biomarker for cancer prognosis.

### 3.4 Comparison of SLCFPS with other glioma prognostic signatures

**FIGURE 4:**
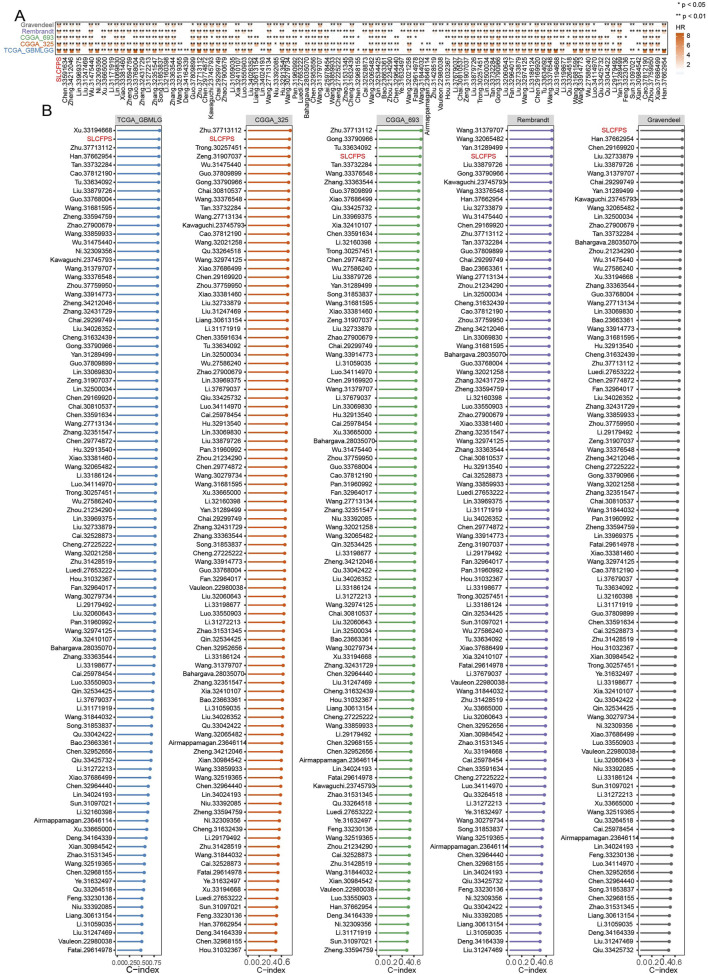
Comparison of SLCFPS with other published glioma signatures. **(A)** Comparison of the hazard ratio (HR) between SCLFPS and other published glioma signatures. **(B)** Comparison of the C-index between SCLFPS and other published glioma signatures.

To determine the predictive robustness of SLCFPS, we systematically compared its prognostic performance against previously published glioma prognostic signatures. Hazard ratio (HR) comparisons demonstrated that SLCFPS exhibited a higher HR than most established glioma signatures, indicating its strong association with patient survival outcomes (Figure 4A). Additionally, we assessed the C-index of SLCFPS relative to other glioma prognostic models. SLCFPS consistently outperformed the majority of existing signatures across glioma cohorts, demonstrating superior prognostic accuracy (Figure 4B). These findings support SLCFPS as a robust and reliable prognostic biomarker for glioma.

### 3.5 Enrichment pathway analysis of the high- and low- SLCFPS groups

**FIGURE 5:**
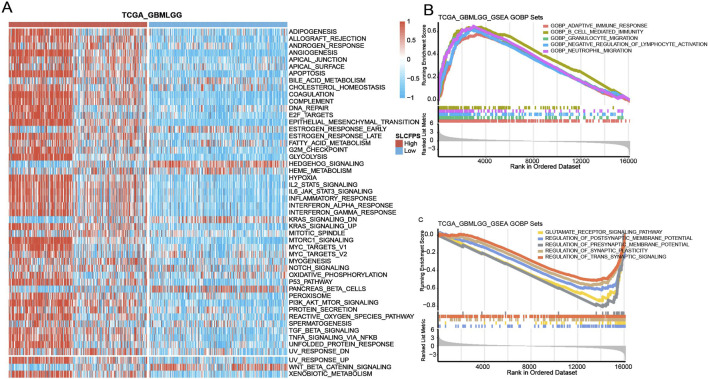
Enrichment pathway analysis of the high and low SLCFPS groups. **(A)** GSVA analysis was conducted to evaluate the scores of each sample in the Hallmark gene sets. Samples were categorized into high-expression and low-expression groups based on SLCFPS, and the differences in Hallmark gene set scores between the two groups were compared. **(B)** The top five Gene Ontology (GO)-enriched pathways in the high SLCFPS group were identified. **(C)** The top five GO-enriched pathways in the low SLCFPS group were identified.

To explore the potential biological mechanisms underlying SLCFPS-associated tumor phenotypes, we performed Gene Set Variation Analysis (GSVA) to compare pathway activity between high and low SLCFPS groups. Hallmark gene set enrichment analysis revealed significant differences in pathway activation between the two groups (Figure 5A), suggesting distinct molecular characteristics. Further Gene Ontology (GO) enrichment analysis identified the top five enriched biological pathways in each group. The high SLCFPS group exhibited significant enrichment in pathways related to tumor progression, immune modulation, and metabolic reprogramming (Figure 5B), while the low SLCFPS group was predominantly enriched in pathways associated with neuronal function and cellular homeostasis (Figure 5C). These findings suggest that SLCFPS may influence glioma progression by modulating key oncogenic and metabolic pathways.

### 3.6 Immune landscape of the high and low SLCFPS groups

**FIGURE 6:**
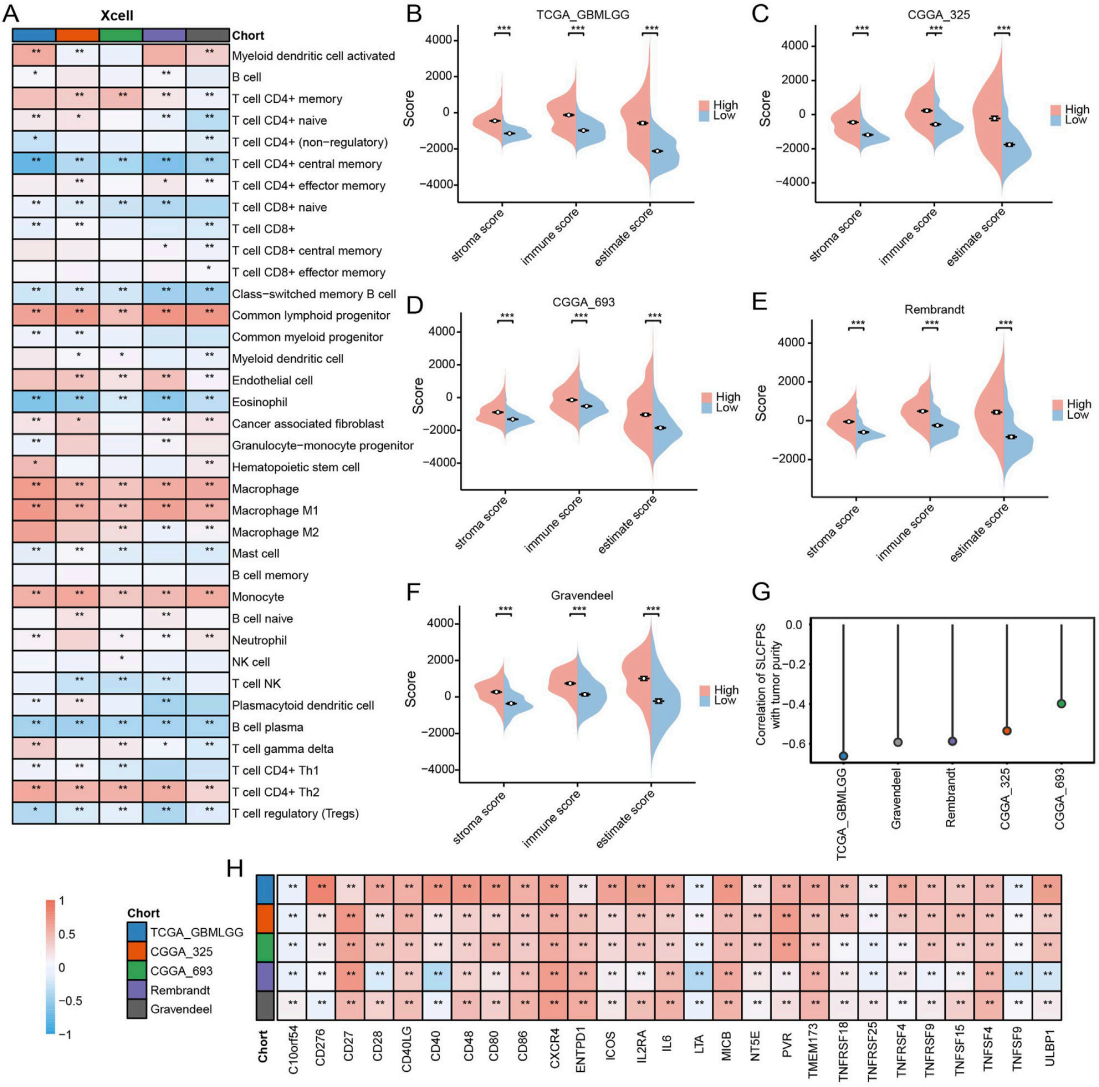
Immune landscape of the high and low SLCFPS groups. **(A)** Correlation and comparison of immune scores calculated using the SLCFPS and xCell methods across five glioma cohorts. **(B)** Comparison of immune scores between high and low SLCFPS expression groups in the TCGA_GBMLGG cohort, analyzed using the ESTIMATE method. **(C)** Comparison of immune scores between high and low SLCFPS expression groups in the CGGA_325 cohort, analyzed using the ESTIMATE method. **(D)** Comparison of immune scores between high and low SLCFPS expression groups in the CGGA_693 cohort, analyzed using the ESTIMATE method. **(E)** Comparison of immune scores between high and low SLCFPS expression groups in the Rembrandt cohort, analyzed using the ESTIMATE method. **(F)** Comparison of immune scores between high and low SLCFPS expression groups in the Gravendeel cohort, analyzed using the ESTIMATE method. **(G)** Comparison of tumor purity correlation calculated using the SLCFPS and ESTIMATE methods across five glioma cohorts. **(H)** Boxplot of the relative expression levels of immune checkpoints in the high and low SLCFPS expression groups. *p < 0.05, **p < 0.01, ***p < 0.001.

To investigate the association between SLCFPS and the tumor immune microenvironment, we analyzed immune scores across five glioma cohorts using multiple computational methods. SLCFPS-derived immune scores exhibited strong correlations with those obtained using the xCell method across all cohorts (Figure 6A), supporting the reliability of our immune profiling approach. Next, we compared immune infiltration levels between high and low SLCFPS groups using the ESTIMATE algorithm. Significantly higher immune scores were observed in the high SLCFPS group across all cohorts, including TCGA_GBMLGG (Figure 6B), CGGA_325 (Figure 6C), CGGA_693 (Figure 6D), Rembrandt (Figure 6E), and Gravendeel (Figure 6F), indicating that high SLCFPS expression is associated with increased immune infiltration. Furthermore, tumor purity analysis demonstrated a strong negative correlation between SLCFPS scores and tumor purity as estimated by the ESTIMATE method across the five glioma cohorts (Figure 6G), suggesting that higher SLCFPS expression is linked to a more immune-enriched tumor microenvironment. Finally, we analyzed the expression levels of immune checkpoint molecules between the high and low SLCFPS groups. Key immune checkpoints, including PD-1, PD-L1, and CTLA-4, were significantly upregulated in the high SLCFPS group (Figure 6H), highlighting the potential relevance of SLCFPS in immunotherapy responsiveness.

### 3.7 Predictive value of SLCFPS for immunotherapy

**FIGURE 7:**
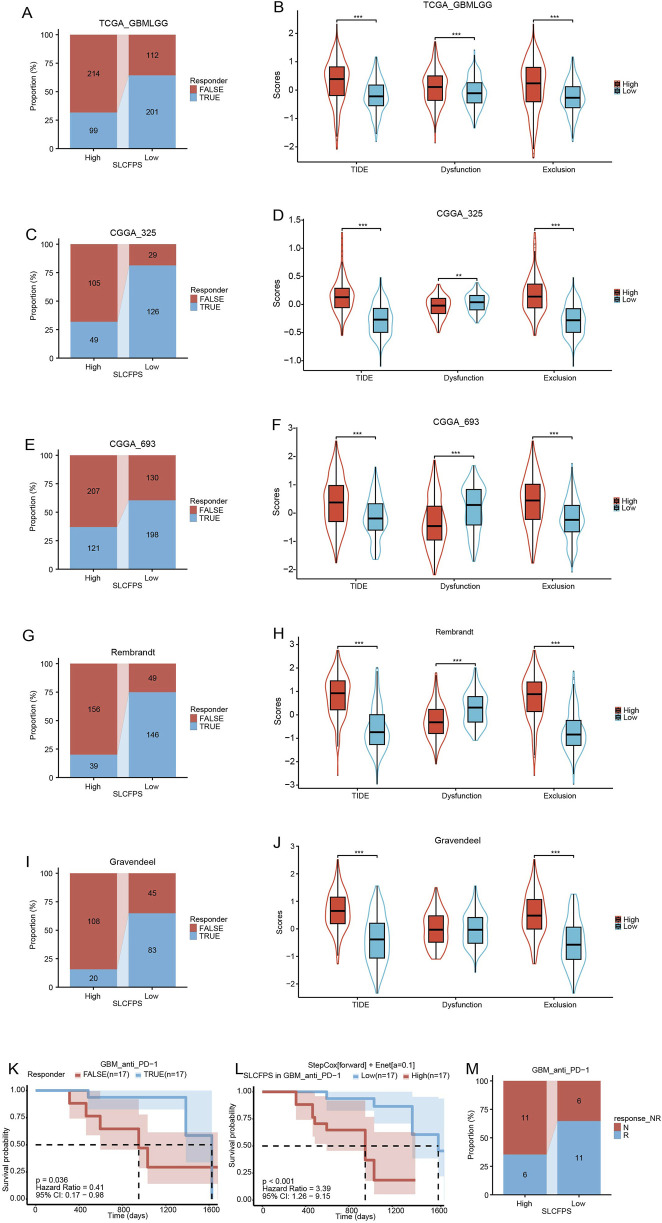
Predictive value of SLCFPS for immunotherapy **(A)** Percentage of immune therapy response predicted by Tumour Immune Dysfunction and Exclusion (TIDE) in the high and low SLCFPS groups within the TCGA_GBMLGG cohort. **(B)** Boxplot of TIDE scores between the high and low SLCFPS expression groups in the TCGA_GBMLGG cohort. **(C)** Percentage of immune therapy response predicted by TIDE in the high and low SLCFPS groups within the CGGA_325 cohort. **(D)** Boxplot of TIDE scores between the high and low SLCFPS expression groups in the CGGA_325 cohort. **(E)** Percentage of immune therapy response predicted by TIDE in the high and low SLCFPS groups within the CGGA_693 cohort. **(F)** Boxplot of TIDE scores between the high and low SLCFPS expression groups in the CGGA_693 cohort. **(G)** Percentage of immune therapy response predicted by TIDE in the high and low SLCFPS groups within the Rembrandt cohort. **(H)** Boxplot of TIDE scores between the high and low SLCFPS expression groups in the Rembrandt cohort. **(I)** Percentage of immune therapy response predicted by TIDE in the high and low SLCFPS groups within the Gravendeel cohort. **(J)** Boxplot of TIDE scores between the high and low SLCFPS expression groups in the Gravendeel cohort. **(K)** Survival analysis of OS in relation to immune therapy response in the PRJNA482620 cohort. **(L)** Survival analysis of OS in high and low SLCFPS expression groups in the PRJNA482620 cohort. **(M)** Percentage of immune therapy response in high and low SLCFPS expression groups in the PRJNA482620 cohort.

To evaluate the potential of SLCFPS as a predictive biomarker for immunotherapy response, we utilized the Tumor Immune Dysfunction and Exclusion (TIDE) algorithm to estimate the likelihood of response to immune checkpoint blockade across five glioma cohorts. A significantly higher proportion of patients in the low SLCFPS group were predicted to respond to immunotherapy compared to those in the high SLCFPS group across all cohorts, including TCGA_GBMLGG (Figure 7A), CGGA_325 (Figure 7C), CGGA_693 (Figure 7E), Rembrandt (Figure 7G), and Gravendeel (Figure 7I). Moreover, TIDE scores were significantly lower in the low SLCFPS group compared to the high SLCFPS group across these cohorts (Figures 7B, D, F, H, J), suggesting that patients with low SLCFPS expression may exhibit greater sensitivity to immune checkpoint inhibitors.

To further validate this observation, we examined a publicly available immunotherapy-treated cohort (PRJNA482620). Survival analysis demonstrated that patients who responded to immunotherapy exhibited significantly longer overall survival (OS) compared to non-responders (Figure 7K). Additionally, patients with low SLCFPS expression had a significantly better OS compared to those with high SLCFPS expression (Figure 7L), consistent with our findings from the TIDE analysis. Notably, a higher proportion of patients in the low SLCFPS group were classified as immunotherapy responders (Figure 7M), further supporting the potential clinical utility of SLCFPS as a predictor of immunotherapy efficacy.

### 3.8 Effectiveness of SLCFPS in predicting drug sensitivity

**FIGURE 8:**
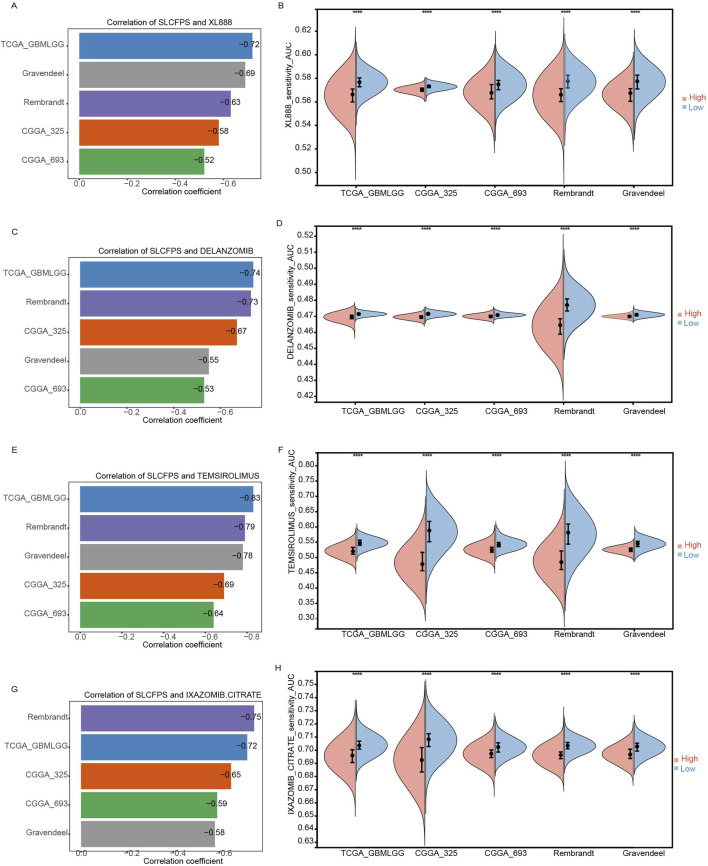
Effectiveness of SLCFPS in predicting drug sensitivity. **(A)** Comparison of the correlation between XL888 drug response scores and SLCFPS scores across five glioma cohorts. **(B)** Comparison of XL888 drug response scores between high and low SLCFPS expression groups across five glioma cohorts. **(C)** Comparison of the correlation between DELANZOMIB drug response scores and SLCFPS scores across five glioma cohorts. **(D)** Comparison of DELANZOMIB drug response scores between high and low SLCFPS expression groups across five glioma cohorts. **(E)** Comparison of the correlation between TEMSIROLIMUS drug response scores and SLCFPS scores across five glioma cohorts. **(F)** Comparison of TEMSIROLIMUS drug response scores between high and low SLCFPS expression groups across five glioma cohorts. **(G)** Comparison of the correlation between IXAZOMIB-CITRATE drug response scores and SLCFPS scores across five glioma cohorts. **(H)** Comparison of IXAZOMIB-CITRATE drug response scores between high and low SLCFPS expression groups across five glioma cohorts.

Given the critical need for personalized treatment strategies in glioma, identifying biomarkers that can predict drug sensitivity is essential for optimizing therapeutic outcomes. Since SLCFPS has shown prognostic potential in glioma, we sought to determine whether it could also serve as a predictive marker for drug response. To investigate the potential utility of SLCFPS in predicting drug sensitivity, we analyzed the correlation between SLCFPS scores and drug response scores across five independent glioma cohorts. Four small-molecule compounds (XL888, Delanzomib, Temsirolimus, and Ixazomib-citrate) exhibited a negative correlation (correlation coefficient < −0.5) with SLCFPS across all cohorts, suggesting that patients with lower SLCFPS expression may exhibit greater sensitivity to these drugs. Specifically, a strong negative correlation was observed between SLCFPS scores and XL888 drug response scores across all five cohorts (Figure 8A), and drug sensitivity analysis revealed that **patients in the low SLCFPS group had significantly lower XL888 response scores compared to those in the high SLCFPS group (Figure 8B), indicating enhanced drug sensitivity in the low-risk group. Similar trends were observed for Delanzomib (Figures 8C, D), Temsirolimus (Figures 8E, F), and Ixazomib-citrate (Figures 8G, H), further supporting the potential role of SLCFPS in predicting patient-specific drug responses. These findings highlight SLCFPS as a promising biomarker for guiding personalized treatment strategies, particularly in identifying glioma patients who may benefit from specific small-molecule inhibitors.

## 4 Discussion

In this study, we developed and validated SLCFPS for glioma by integrating multi-cohort survival analysis and machine learning-based predictive modeling. Our results demonstrate that SLCFPS is a robust prognostic biomarker, effectively stratifying glioma patients into high- and low-risk groups with significant differences in overall survival. Furthermore, SLCFPS exhibited superior predictive performance compared to previously published glioma prognostic models. Beyond its prognostic utility, SLCFPS was strongly associated with tumor immunity, immunotherapy response, and drug sensitivity, underscoring its potential role in guiding personalized treatment strategies.

Gliomas exhibit significant molecular and clinical heterogeneity, current bio-markers alone fail to capture the full complexity of glioma progression (Mathur et al., 2024). By leveraging machine learning and survival analysis across multiple cohorts, we identified a prognostic model based on SLC family genes that outperforms existing glioma signatures. The superior predictive power of SLCFPS is attributed to the critical roles of SLC proteins in tumor metabolism, nutrient transport, and cellular homeostasis. Many SLC family members have been implicated in glioma biology, regulating processes such as glucose and amino acid metabolism, oxidative stress response, and resistance to apoptosis. Given that metabolic reprogramming is a hallmark of glioma (Kadiyala et al., 2021), the integration of SLC genes into a prognostic model provides a biologically relevant approach to risk stratification.

The tumor immune microenvironment plays a crucial role in glioma progression and therapeutic resistance (Jayaram and Phillips, 2024). Our study revealed that high SLCFPS expression correlates with an immunosuppressive tumor microenvironment, characterized by increased immune infiltration and upregulation of immune checkpoint molecules such as PD-1, PD-L1, and CTLA-4. These findings suggest that patients with high SLCFPS scores may exhibit immune evasion mechanisms, potentially leading to resistance to conventional immunotherapies. Further immune landscape analysis demonstrated that high SLCFPS expression was associated with higher immune scores across multiple glioma cohorts, as assessed by the ESTIMATE and xCell algorithms. Interestingly, while high immune scores often indicate an active immune response, gliomas are known to exhibit a paradoxical immune-suppressive phenotype, wherein infiltrating immune cells fail to mount an effective anti-tumor response. This suggests that SLCFPS may serve as a biomarker for gliomas with immune dysfunction, guiding the selection of appropriate immunotherapeutic strategies.

With the increasing clinical focus on immune checkpoint blockade therapies, reliable biomarkers are needed to predict patient response. Our analysis using the TIDE algorithm revealed that patients with low SLCFPS expression were more likely to respond to immune checkpoint inhibitors (ICIs). Additionally, in the PRJNA482620 immunotherapy-treated cohort, patients with low SLCFPS expression exhibited significantly longer overall survival, further supporting the predictive value of SLCFPS for immunotherapy outcomes. These findings suggest that SLCFPS could be used to identify glioma patients who are more likely to benefit from immune checkpoint blockade therapies, thereby optimizing treatment selection.

In addition to its prognostic and immunological relevance, SLCFPS was significantly correlated with drug sensitivity to multiple small-molecule inhibitors. Patients with low SLCFPS expression demonstrated greater sensitivity to compounds such as XL888, Delanzomib, Temsirolimus, and Ixazomib-citrate, suggesting that SLCFPS may serve as a predictive biomarker for targeted therapies. These drugs are known to target key oncogenic pathways, including HSP90 inhibition (XL888) (Mansfield et al., 2024), proteasome inhibition (Delanzomib, Ixazomib-citrate) (Kegyes et al., 2023), and mTOR inhibition (Temsirolimus) (Gupta et al., 2024).The observed correlation between SLCFPS and drug sensitivity highlights its potential utility in personalized therapy selection. Future studies should explore the mechanistic basis of these drug interactions, as well as the feasibility of incorporating SLCFPS into clinical decision-making for glioma treatment.

Despite the promising findings, this study has several limitations. First, our analysis is retrospective and relies on publicly available datasets, which may introduce selection bias and data heterogeneity. The inherent differences in sequencing platforms, patient population characteristics, and data preprocessing methods across datasets could potentially impact the generalizability of our findings. Second, although SLCFPS was validated across multiple independent cohorts, prospective clinical validation in larger, well-controlled clinical studies is necessary before its routine application in clinical practice. Third, while our study provides strong bioinformatic and statistical evidence, the biological mechanisms linking SLC proteins to glioma progression and therapy resistance remain incompletely understood. Functional experiments are needed to elucidate the roles of individual SLC genes in glioma pathogenesis and their interactions with the tumor microenvironment. Finally, although we demonstrated an association between SLCFPS and immunotherapy response, the predictive value of SLCFPS in guiding immunotherapy and drug selection should be validated in prospective clinical trials. Future studies should also investigate whether integrating SLCFPS with other molecular biomarkers can further enhance its prognostic and predictive accuracy.

## 5 Conclusion

In this study, we developed and validated SLCFPS as a novel prognostic biomarker for glioma, demonstrating its strong predictive value for patient survival. SLCFPS effectively stratifies patients into high- and low-risk groups and outperforms existing biomarkers. It also correlates with immune microenvironment changes, such as increased immune infiltration and immune checkpoint molecule upregulation, suggesting its potential role in identifying immune-suppressive glioma subtypes and predicting immunotherapy responsiveness. Additionally, SLCFPS was linked to drug sensitivity, highlighting its relevance for personalized treatment. While further validation is needed, SLCFPS shows promise for improving prognosis, therapy selection, and patient outcomes in glioma.

## Data Availability

The clinical data and RNA-seq data for the five glioma cohorts were downloaded from the Gliovis website (GlioVis - Visualization Tools for Glioma Datasets). The immunotherapy data for GBM (PRJNA482620) was obtained from the TIGER database http://tiger.canceromics.org/#/download. The SLC family gene set was collected from the HGNC website (Home HUGO Gene Nomenclature Committee). The original contributions presented in the study are included in the article/supplementary material, further inquiries can be directed to the corresponding authors.
